# Single, Double and Quadruple Alanine Substitutions at Oligomeric Interfaces Identify Hydrophobicity as the Key Determinant of Human Neutrophil Alpha Defensin HNP1 Function

**DOI:** 10.1371/journal.pone.0078937

**Published:** 2013-11-13

**Authors:** Le Zhao, W. David Tolbert, Bryan Ericksen, Changyou Zhan, Xueji Wu, Weirong Yuan, Xu Li, Marzena Pazgier, Wuyuan Lu

**Affiliations:** 1 Translational Medicine Center, the First Affiliated Hospital, School of Medicine, Xi’an Jiaotong University, Xi’an, China; 2 Institute of Human Virology and Department of Biochemistry and Molecular Biology, University of Maryland School of Medicine, Baltimore, Maryland, United States of America; Institute of Molecular Genetics IMG-CNR, Italy

## Abstract

HNP1 is a human alpha defensin that forms dimers and multimers governed by hydrophobic residues, including Tyr^16^, Ile^20^, Leu^25^, and Phe^28^. Previously, alanine scanning mutagenesis identified each of these residues and other hydrophobic residues as important for function. Here we report further structural and functional studies of residues shown to interact with one another across oligomeric interfaces: I20A-HNP1 and L25A-HNP1, plus the double alanine mutants I20A/L25A-HNP1 and Y16A/F28A-HNP1, and the quadruple alanine mutant Y16A/I20A/L25A/F28A-HNP1. We tested binding to HIV-1 gp120 and HNP1 by surface plasmon resonance, binding to HIV-1 gp41 and HNP1 by fluorescence polarization, inhibition of anthrax lethal factor, and antibacterial activity using the virtual colony count assay. Similar to the previously described single mutant W26A-HNP1, the quadruple mutant displayed the least activity in all functional assays, followed by the double mutant Y16A/F28A-HNP1. The effects of the L25A and I20A single mutations were milder than the double mutant I20A/L25A-HNP1. Crystallographic studies confirmed the correct folding and disulfide pairing, and depicted an array of dimeric and tetrameric structures. These results indicate that side chain hydrophobicity is the critical factor that determines activity at these positions.

## Introduction

Defensins are a family of antimicrobial peptides of innate immunity broadly active against bacteria, viruses and toxins [1], [2], [3], [4], [5], [6]. They are small (2–5 kDa), stabilized structurally by three disulfide bonds, form oligomers, and are divided into α [6], β [7] and θ [8] structural classes. There are six human α-defensins: human neutrophil peptides (HNP) 1–4 [9], [10], [11], [12], [13] and human defensins (HD) 5–6 [14], [15]. The structural basis for defensin antimicrobial activity against bacteria and viruses is only partially understood, but it is clear that defensins possess two fundamental characteristics: cationicity and hydrophobicity. α-Defensins are positively charged, and their activity against Gram negative bacteria such as *Escherichia coli* is thought to be mediated by the electrostatic attraction between cationic arginine residues and the anionic phospholipids of bacterial membranes [16]. By contrast, the human α-defensins are more selective for Gram positive strains, and specific, chiral interactions with lipid II molecules have been proposed to be primarily responsible for activity against Gram positive bacterium, *Staphylococcus aureus* [17], as is the case with several other defensins [18], [19], [20], [21]. Defensins provide two types of protection against bacilli: they are active against *Bacillus cereus* directly with a high degree of potency [22], [23], and they bind to and inhibit bacterial toxins such as anthrax lethal factor (LF) [24], [25], [26], [27], [28]. A picture of the myriad functions of HNP1 and HD5 is emerging that identifies hydrophobicity as more critical than cationicity, which can be explained partially by the hydrophobic effect on the α-defensin “canonical” dimer. For HNP1, the canonical dimer interface is formed by the antiparallel extensions of the β2 strands stabilized by the reciprocal main-chain hydrogen bonds contributed by Thr^18^ and Ile^20^, and the hydrophobic packing of the side chains of Tyr^16^, Tyr^21^, Phe^28^ and the Cys^2^–Cys^30^ disulfide [27], [29]. Underlying these hydrophobic contacts are the aromatic rings of Trp^26^ [28], constituting the bulk of the hydrophobic core of the monomer.

**Figure 1:**
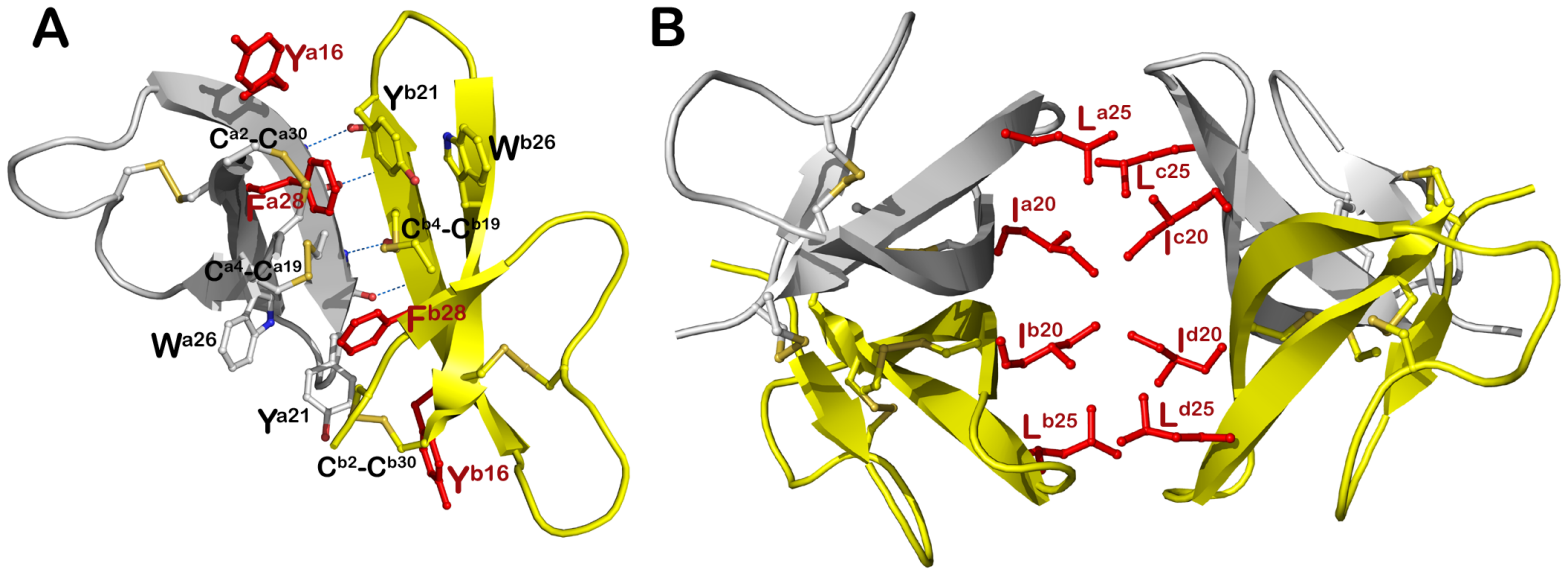
Quaternary structure of wild type HNP1. (A) Dimeric and (B) tetrameric assembly of HNP1 in a crystal (PDB:3GNY, [27]). Disulfide bonds and residues involved in oligomerization are shown as balls and sticks and reciprocal main chain hydrogen bonds are shown as blue dashes. Residues subjected to Ala-substitutions in presented studies are highlighted in red.

Alanine scanning mutagenesis is a powerful tool for exploring the contributions of individual side chains to aspects of protein and peptide function such as binding [30], [31], [32], stability [33], [34], [35], and catalysis [36], [37]. Alanine scanning mutageneses of both HNP1 [28] and HD5 [26] identified hydrophobic residues as the most important for activity, especially HNP1 Trp^26^ and HD5 Leu^29^. In the alanine scan of HNP1, the hydrophobic residues of Tyr^16^, Ile^20^, Leu^25^ and Phe^28^ were also shown to be significant, since the Y16A, I20A, L25A and F28A mutants all had decreased bactericidal activity against *S. aureus* [28]. Surface plasmon resonance (SPR) studies supported the antibacterial assays, identifying F28A-HNP1 as the second lowest self-association on the HNP1 surface of all of the alanine scanning mutants, which corresponded to its second worst antibacterial activity, after W26A. However, in spite of the power of alanine scanning, it can generate misleading results when at least two side chains interact, which can cause non-additivity [38], [39]. In order to circumvent this potential shortcoming and shed light upon the cumulative effects of side chain interactions, if any, multiple mutants can be constructed. Crystallography identified two pairs of side chains that would be particularly insightful: Tyr^16^/Phe^28^ and Ile^20^/Leu^25^. Tyr^16^ and Phe^28^ mapped to the dimer interface, making side chain contacts with the opposing monomer. The canonical dimer interface of HNP1 is formed by the antiparallel extensions of the β2 strands stabilized by the reciprocal main-chain hydrogen bonds contributed by Thr^18^ and Ile^20^, and the hydrophobic packing of the side chains of Tyr^16^, Tyr^21^, Trp^26^, Phe^28^ and the Cys^2^–Cys^30^ and Cys^4^–Cys^19^ disulfides (Fig. 1A) [27], [28]. Further analysis of probable quaternary structures by the Protein Interfaces, Surfaces and Assemblies (PISA) software indicates that the HNP1 dimer forms a tetramer or dimer of dimers in a crystal. The dimer-dimer interface consists of the Ile^20^ and Leu^25^ side chains and the assembly is maintained exclusively through van der Waals interactions (Fig. 1B).

Here we report the x-ray crystal structures of I20A-HNP1, I20A/I25A-HNP1, Y16A/F28A-HNP1 and Y16A/I20A/L25A/F28A-HNP1. The functional consequences of each mutation are also investigated using SPR, fluorescence polarization, an enzyme kinetic assay to quantify LF inhibition, and the virtual colony count assay to quantify antibacterial activity.

## Materials and Methods

### Synthesis and Folding of Defensins

All five HNP1 mutants (Table 1) were synthesized on an ABI 433A synthesizer using an optimized HBTU activation/DIEA *in situ* neutralization protocol developed by Kent and coworkers for Boc chemistry solid phase peptide synthesis (SPPS) [40]. All peptides were purified by C_18_ reversed phase high performance liquid chromatography (RP-HPLC), and their molecular masses ascertained by electrospray ionization mass spectrometry (ESI-MS) (data not shown). Correct folding of the Y16A/F28A, I20A/L25A, I20A, and L25A mutants was achieved at 0.25 mg/mL in 25% *N,N*-dimethylformamide, 2 M urea, 50 mM Tris/HCl, 3 mM reduced and 0.3 mM oxidized glutathione (pH 8.3) overnight at room temperature [41]. Y16A/I20A/L25A/F28A-HNP1, with the side chains of Cys^9^ and Cys^29^ acetamidomethyl (Acm) protected, was first oxidized at 0.25 mg/mL in 50 mM Tris/HCl buffer (pH 8.3) by stirring in the open air overnight to form two disulfide bridges, Cys^2^–Cys^30^ and Cys^4^–Cys^19^. The desired folding intermediate, confirmed by disulfide mapping through digesting with 0.1 mg/mL chymotrypsin for 1.5 hr at room temperature in 50 mM Tris/HCl, 20 mM CaCl_2_, 0.005% Triton X-100 (pH 8.3), was then treated by 0.5 mM iodine for 45 min at 0.5 mg/mL in an acidic solution containing 0.1 M citric acid, 0.2 M HCl and 20% methanol to deprotect Acm and form the third disulfide bond. The reaction was quenched by 0.2 M ascorbic acid, and the fully folded peptide was purified to homogeneity by RP-HPLC and its molecular mass verified by ESI-MS. Defensin stock solutions prepared with water were quantified spectroscopically at 280 nm using molar extinction coefficients calculated according to the algorithm of Pace *et al* [42].

**Table 1:** The amino acid sequences of wild type HNP1 and HNP1 analogs.

HNP1	ACYCRIPACI^10^ AGERRYGTCI^20^ YQGRLWAFCC^30^
Y16A/F28A	ACYCRIPACI^10^ AGERR**A**GTCI^20^ YQGRLWA**A**CC^30^
Y16A/I20A/L25A/F28A	ACYCRIPACI^10^ AGERR**A**GTC**A** ^20^ YQGR**A**WA**A**CC^30^
I20A/L25A	ACYCRIPACI^10^ AGERRYGTC**A** ^20^ YQGR**A**WAFCC^30^
I20A	ACYCRIPACI^10^ AGERRYGTC**A** ^20^ YQGRLWAFCC^30^
L25A	ACYCRIPACI^10^ AGERRYGTCI^20^ YQGR**A**WAFCC^30^

### Crystallization and Data Collection

Lyophilized HNP1 mutant proteins were dissolved in water (20 mg/mL), mixed in a 1∶1 ratio (1 µl total) with appropriate precipitant solutions and left to equilibrate in a hanging drop at room temperature. Crystals were flash frozen in liquid nitrogen after briefly soaking in the crystallization condition plus 15–25% glycerol prior to data collection.

For the I20A-HNP1 and I20A/L25A-HNP1 mutants, data were collected using a rotating anode x-ray generator Rigaku-MSC Micromax 7 and a Raxis-4++ image plate detector (at the X-ray crystallography core facility, University of Maryland, Baltimore). Diffraction data for HNP1 Y16A/F28A and Y16A/I20A/L25A/F28A mutants were collected at the Stanford Synchrotron Radiation Light Source (SSRL) BL7-1 beamline on an ADSC Quantum 315 area detector. All data were processed and reduced with HKL2000 [43]. Structures were solved by molecular replacement with Phaser [44] from the CCP4 suite based on the coordinates of the HNP1 monomer (PDB: 3GNY). Refinement was carried out with Refmac [45] and/or Phenix [46] and model building was done with COOT [47]. Data collection and refinement statistics are shown in Table 2. Ramachandran statistics were calculated with Molprobity [48] and illustrations were prepared with Pymol molecular graphics (http://pymol.org) or Molscript [49].

**Table 2:** Data collection and refinement statistics.

		Crystals of HNP1 mutants						
		I20A	I20A/L25A		Y16A/F28A		Y16A/I20A/L25A/F28A	
Data collection								
Wavelength, Å		1.54	1.54		0.98		1.00	
Space group		P4_1_	C2		H3		H3	
Cell parameters								
a, b, c, Å		37.7, 37.7, 40.5	75.0, 62.6, 42.5		88.6, 88.6, 54.1		83.8, 83.8, 51.3	
α, β, γ, °		90, 90, 90	90, 99.7, 90		90, 90, 120		90, 90, 120	
Molecules/a.u.		2	8		4		4	
Resolution, (Å)^a^		50-1.7 (1.73-1.7)	50-1.66 (1.69-1.66)		50-2.0 (2.03-2.00)		50.0-1.9 (1.93-1.90)	
# of reflections								
Total		17,297	73,805		58,691		60,651	
Unique		8,953	22,465		10,671		10,457	
R_merg_ b, %		13.9 (67.4)	8.1 (44.8)		10.3 (75.9)		3.6 (96.0)	
I/σ		12.7 (2.0)	16.5 (1.6)		21.6 (1.3)		56 (2.2)	
Completeness, %		100 (99.0)	98.3 (84.1)		99.5 (98.5)		99.1 (100)	
Redundancy		4.8 (3.8)	2.3 (3.3)		5.5 (4.2)		5.8 (5.8)	
Refinement Statistics								
Resolution, Å	27.6-1.72			20-1.70		18.0-2.00		24.2-1.90
R^c^, %	20.1			18.5		19.0		19.9
R_free_ d, %	22.9			23.1		22.8		23.6
# of atoms								
Protein	470			1,815		900		876
Water	26			209		8		8
Ligand/Ion	6			6		0		0
Overall B value (Å)^2^								
Protein	38.2			28.6		64.8		72.7
Water	46.3			37.8		60.8		67.0
Ligand/Ion	67.6			20.9		–		–
Root mean square deviation								
Bond lengths, Å	0.015			0.024		0.018		0.007
Bond angles,°	1.6			1.9		1.9		1.33
Ramachandran^e^								
favored, %	94.6			96.8		96.4		94.4
allowed, %	5.4			3.2		3.6		3.6
outliers, %	0.0			0.0		0.0		0.0
PDB code	4LBB			4LBF		4LB1		4LB7

a Values in parentheses are for highest-resolution shell.

b *R*_merge_ = ∑|*I* - <*I*>|/∑*I*, where *I* is the observed intensity and <*I*> is the average intensity obtained from multiple observations of symmetry-related reflections after rejections.

c *R* = ∑||F_o_|- | F_c_||/∑|F_o_ |, where F_o_ and F_c_ are the observed and calculated structure factors, respectively.

d R_free_ = defined by by Brünger [68].

e Calculated with MolProbity [48].

### SPR Based wt-HNP1 and gp120 Binding

Experiments were performed at 25°C on a BIAcore T100 System (BIAcore, Inc., Piscataway, NY). The assay running buffer was 10 mM HEPES, 150 mM NaCl, 0.05% surfactant P20, pH 7.4 (±3 mM EDTA). 233 response units (RU) of HNP1 or 2770 RU of gp120 were immobilized on CM5 sensor chips using the amine-coupling chemistry. Analytes were introduced into the flow-cells at 30 µl/min in the running buffer. Association and dissociation were assessed for 5 and 10 min, respectively. After each analysis, the sensor chip surfaces were regenerated with 30 mM HCl for HNP1, 10 mM glycine solution (pH 2.0) and 10 mM NaOH for gp120, and equilibrated with the running buffer before the next injection. Binding isotherms were analyzed with BIAevaluation software.

### Fluorescence Polarization-based Defensin and N36-gp41 Binding

An N-terminally acetylated N36 (HIV-1 gp160 546–581) peptide derived from the N-terminus of gp41 was synthesized by Boc-chemistry SPPS and purified to homogeneity by preparative C18 RP-HPLC. Succinimidyl ester-activated carboxyfluorescein (FAM-NHS) was covalently conjugated to N-acetylated N36 peptide via its Lys^574^ (HIV gp160 numbering) side chain in DMF, and the resultant product N-acetyl-N36-FAM was HPLC-purified and lyophilized. The defensin-N36 binding experiments were performed in 384-well plates on a Tecan Infinite M1000 multimode plate reader. 2-fold serially diluted defensins were prepared in PBS and incubated with 50 nM FAM-labeled N36 in a total volume of 100 µl per well. After a 30 min incubation at room temperature, fluorescence polarization values were measured at exciting and emitted wavelengths of 470 nm and 530 nm, respectively.

### LF inhibition kinetics

A 2-fold dilution series of defensin, ranging from 1024 to 1 nM in 20 mM HEPES buffer containing 1 mM CaCl_2_ and 0.5% Nonidet P-40 (pH 7.2), was incubated at 37°C for 30 min with 1 µg/mL (∼10 nM) of LF. Then, 20 µl of 1 mM LF substrate was added into each well to a final concentration of 100 µM in a total volume of 200 µl. Kinetic measurements of LF enzymatic activity were monitored at 405 nm over 30 min at 37°C on a Tecan Infinite M1000 microplate reader. Data were presented in a plot showing percent inhibition *versus* defensin concentration, from which IC_50_ values (the concentration of defensin that reduced the enzymatic activity of LF by 50%) were derived by a non-linear regression analysis [27], [28].

### Virtual Colony Count

Antibacterial assays against *Escherichia coli* strain American Type Culture Collection (ATCC) 25922, *Staphylococcus aureus* ATCC 29213, and *Bacillus cereus* ATCC 10876 were conducted using the Virtual Colony Count 96-well kinetic turbidimetric method [22]. Strains were obtained from ATCC (Manassas, VA). A 2-fold dilution series of defensin, ranging from 256 to 1 µg/mL, was incubated with ∼5×10^5^ virtual colony forming units (CFUv)/mL bacteria at 37°C for 2 h in 10 mM sodium phosphate buffer, pH 7.4, 1% tryptic soy broth (TSB), followed by addition of twice-concentrated Mueller-Hinton broth and kinetic measurements of bacterial growth at 650 nm every 5 minutes over 12 h using a Tecan Infinite M1000 plate reader set to shake 3s orbitally before each read. *B. cereus* was also assayed using a 2-fold dilution series of defensin ranging from 4-0.016 µg/mL. The 10 mM sodium phosphate incubation buffer included 1% TSB to increase the sensitivity of bacteria to defensin activity [9]. The zero time point was discarded and the optical density at the 5-minute time point was subtracted from subsequent growth kinetic optical density readings, as previously described [23]. Sextuplicate calibration curves were measured at a threshold change in optical density at 650 nm of 0.05. The virtual LD_50_ (vLD_50_), vLD_90_, vLD_99_, and vLD_99.9_ were reported as the defensin concentration that resulted in survival rates of 0.5, 0.1, 0.01, and 0.001, respectively. Measurements were done in triplicate on three separate days, except the assay of Y16A/I20A/L25A/F28A-HNP1 against *B. cereus* from 256 to 1 µg/mL, which was done in triplicate on the same 96-well plate. Statistical p-values were calculated as the output of the paired two-tailed Microsoft Excel T.TEST function.

## Results

### Total Chemical Synthesis and Oxidative Folding of HNP1 Analogs

Crude HNP1 mutants, after HF cleavage and ether precipitation, gave rise to molecular masses in agreement with the expected values calculated on the basis of the average isotopic compositions of reduced defensins. Using the efficient protocol established for folding of HNP1, the reduced analogs Y16A/F28A-HNP1, I20A/L25A-HNP1, I20A-HNP1, and L25A-HNP1 productively folded in the presence of 2 M urea and 25% DMF, giving the three disulfide bridges of Cys^2^–Cys^30^, Cys^4^–Cys^19^ and Cys^9^–Cys^29^. For Y16A/I20A/L25A/F28A-HNP1, Cys^9^ and Cys^29^ were strategically selected to be protected by Acm during peptide synthesis. The correctly folded product could then be produced in high yield through a 2-step folding procedure. Two folding species were produced after the first air-oxidation reaction, one of which generated three desired major segments after chymotrypsin digestion: [AC^2^Y][AAC^29^(Acm)C^30^] (found 791.3 Da, calculated 791.9 Da), [QGRAW] (found 617.3 Da, calculated 616.7 Da) and [C^4^RIPAC^9^(Acm)IAGERRAGTC^19^AY] (found 1979.6 Da, calculated 1980.3 Da), confirming the presence of disulfide bridges between Cys^2^–Cys^30^ and Cys^4^–Cys^19^. This folding intermediate was then iodine-oxidized to form the third disulfide bond between Cys^9^ and Cys^29^.

### Crystallographic Monomers of HNP1 Analogs are Similar to the Previously Determined Structure of the HNP1 Monomer

The crystal structures of I20A-HNP1, I20A/L25A-HNP1, Y16A/F28A-HNP1 and Y16A/I20A/L25A/F28A-HNP1 were solved by molecular replacement at resolutions between 1.66–2.0 Å. In each case, multiple copies of defensin molecules were present in the asymmetric unit of the crystal (Table 2). Structural alignment of these crystallographically independent monomers reveals no changes to the core structure, which is the same as in wild type HNP1 (Fig. S1). When superimposed, the root-mean-square (RMS) deviations between 30 equivalent Cα-atoms of mutants and wild-type HNP1 are in the range of 0.51–0.78 Å. There are no changes to the network of three disulfide bridges. Both the disulfide bond connectivity and stereochemistry are identical to wild-type HNP1. In addition to the flexible termini, the major difference is observed for the loop region connecting the second and third β strands. Since the β2–β3 loop of HNP1 is involved in intensive crystal contacts, these differences are attributed to the effect of crystal packing rather than determined by the intrinsic properties of the backbone.

### Mutants Exhibit a Variety of Oligomerization Structures

**Figure 2:**
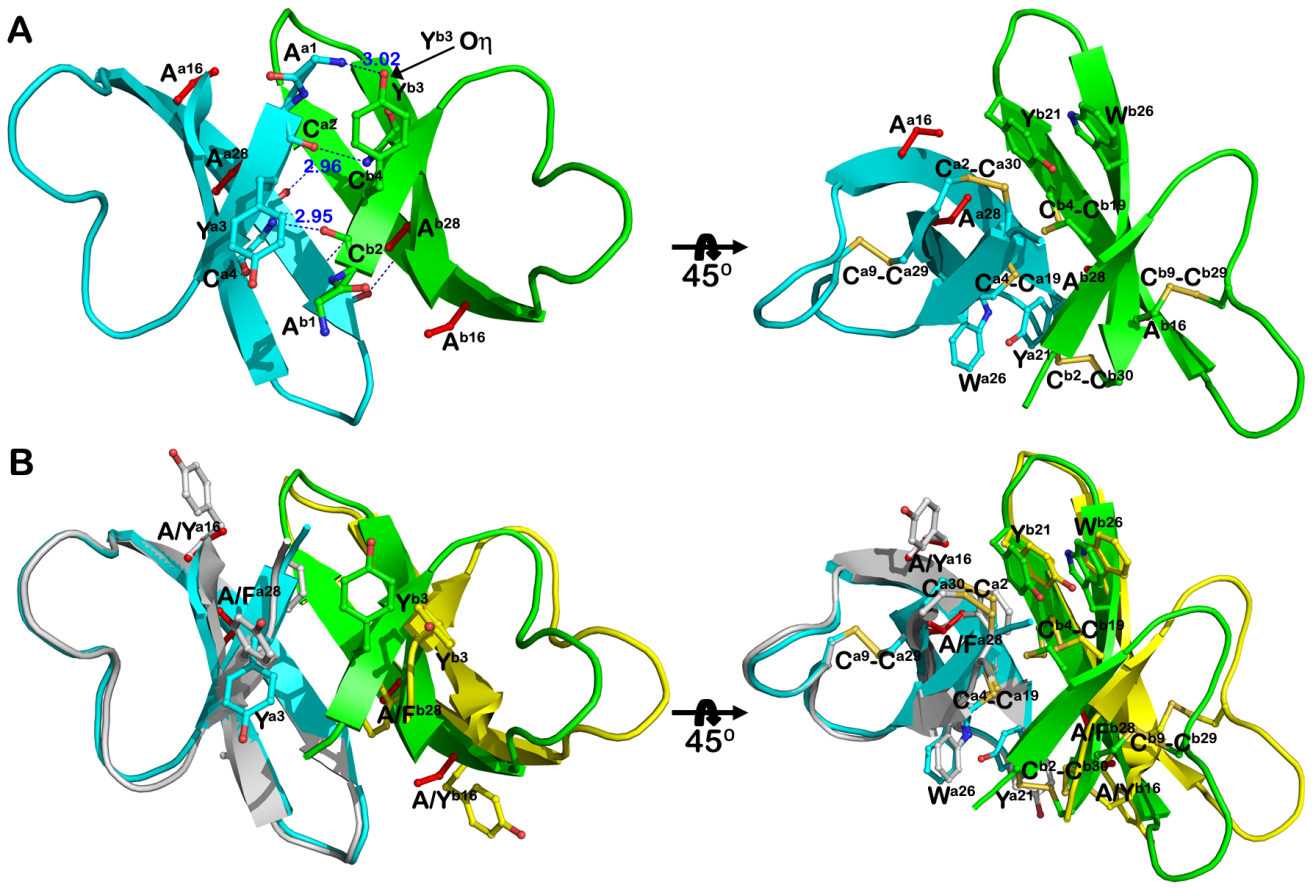
Dimerization of Y16A/F28A-HNP1. (A) Ribbon diagram of the Y16A/F28A HNP1 dimer with hydrophilic (left panel) and hydrophobic (right panel) residues stabilizing the dimer shown as balls and sticks. Mutated residues are shown in red and H-bonds are displayed as blue dashes. In addition to the main chain hydrogen bonds formed between Thr^18^ and Ile^20^, three new hydrogen bonds, between Cys^2^ and Cys^4^ and between the hydroxyl of Tyr^3^ and Ala^1^ help stabilize the opposite face of the dimer. (B) Superposition of Y16A/F28A and wild type HNP1 dimers. Y16A/F28A is in cyan and green and wild type HNP1 in grey and yellow.

Analysis of HNP1 quaternary structures by PISA indicates that Tyr^16^ and Phe^28^ are essential for HNP1 dimerization contributing 16 and 11%, respectively, of the solvent-accessible surface buried by each monomer at the dimer interface. Recently we have shown that single point mutations of Y16A or F28A had no effect on the ability of HNP1 to form stable dimers in crystals [28]. To further test the role of Tyr^16^ and Phe^28^ in mediating the HNP1 dimer, we prepared the double mutant Y16A/F28A-HNP1. Analysis of intermolecular contacts within the Y16A/F28A-HNP1 crystal indicates that mutant monomers assemble into compact dimers reassembling canonical dimers of wild-type HNP1 (Fig. 2). The replacement of the bulky side chains of Tyr^16^ and Phe^28^ by linear Ala side chains allows the dimer to pack more tightly and form an additional set of H-bonds on the opposite face of the dimer. The new set of four H-bonds pins the short β1 strand of one monomer to the equivalent strand of the other monomer to form a two-stranded antiparallel β-sheet (Fig. 2A). These include the reciprocal main-chain H-bonds contributed by the carbonyl oxygen of Cys^2^ and the nitrogen of Cys^4^ and main chain nitrogen of Ala^1^ of monomer a and the hydroxyl of Tyr^3^ of monomer b. Formation of this dimer results in the average burial of more than 495 Å^2^ of molecular surface per each monomer, which compares to an average of 370 Å^2^ for the wild-type HNP1 dimer (PDB:3GNY, [27]). The significant increase in the average value of the molecular surface buried within inter-monomer interactions of Y16A/F28A-HNP1 as compared to the wild-type dimer is attributed to the presence of additional interactions mediated by the β1 strands. Similarly, a more compact dimer was formed by the replacement of the single Phe^28^ at the dimer interface [28]. Superimposition analysis (Fig. S2) of the Y16A/F28A-HNP1 and F28A-HNP1 dimers reveals very close similarity as shown by an average RMDS value of 0.6 Å for 60 aligned Cα atoms and an almost identical molecular surface buried within the dimer (495 and 500 Å^2^ per monomer for the Y16A/F28A-HNP1 and F28A-HNP1 dimer, respectively). Introduction of mutations at the dimer interface have no effect on the ability of Y16A/F28A-HNP1 to form a stable tetramer, which closely resembles the wild type HNP1 tetramer (Fig. S2).

Surprisingly, although the asymmetric unit of the I20A-HNP1 crystal contained two monomers they were not arranged into a dimer. Analysis of intermolecular contacts within the crystal unambiguously rules out the formation of any quaternary structure for the I20A-HNP1 mutant.

**Figure 3:**
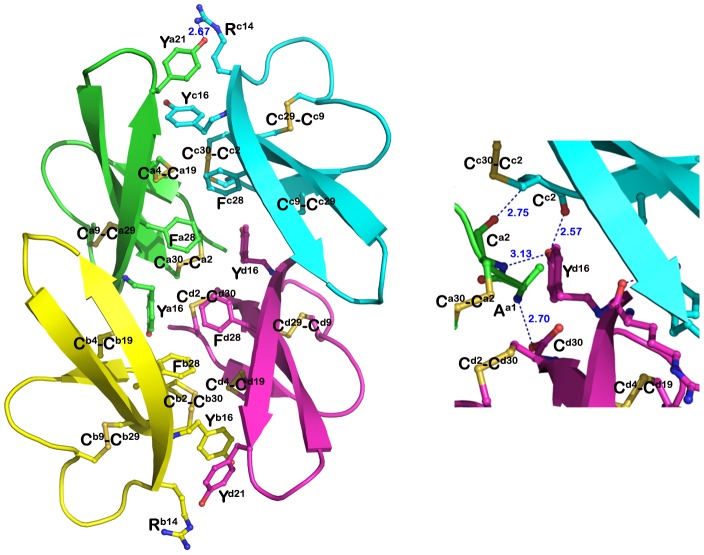
I20A/L25A-HNP1 mutant tetramer. The tetramer is stabilized by intensive hydrophobic interactions contributed mainly by the side chain atoms of Phe^28^ and Tyr^21^ and Cys^4^–Cys^19^ and Cys^2^–Cys^30^ The close-up view shows one of two hydrogen bond networks formed at the tetramer interface. The residues contributing to the tetramer formation are shown as balls and sticks. H-bond distances are given in Angstroms.

**Figure 4:**
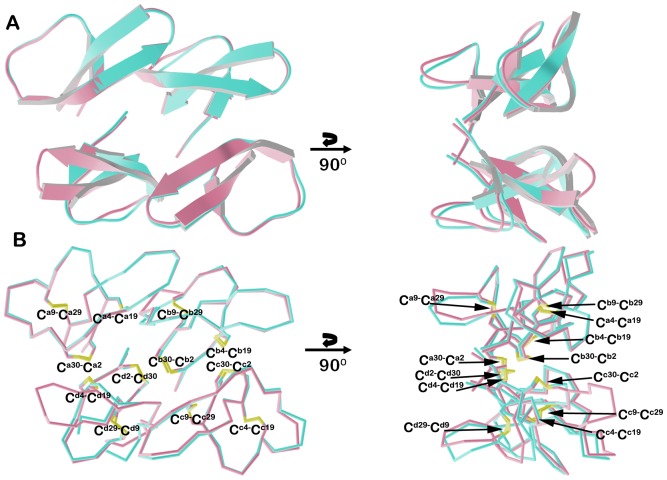
I20A/L25A-HNP1 tetramers. (A) Ribbon and (B) and α-carbon traces of the two independent tetramers of I20A/L25A-HNP1 present in the asymmetric unit of the crystal. Tetramers could be superimposed with the RMSD of 0.744 Å.

We failed to obtain diffracting crystals of the L25A-HNP1 mutant. I20A/L25A-HNP1 crystallizes as a tetramer with two independent tetramers in the asymmetric unit of the crystal (Fig. 3 and Fig. 4). The RMSD between tetramers is 0.744 Å. Unlike wild type HNP1 and most other HNP1 mutants, the tetramer I20A/L25A-HNP1 is not formed as a dimer of dimers. Each monomer contributes equally to tetramer stability with a combined buried surface area of approximately 2760 Å^2^ (as compared to 2280 Å^2^ of the wild type HNP1 tetramer). Tyr^16^ and Phe^28^ make up the bulk of the hydrophobic core with contributions from Tyr^21^ and the adjacent disulfide bonds of Cys^4^–Cys^19^ and Cys^2^–Cys^30^. In addition, there are two core sets of hydrogen bonds in the tetramer center that help in its stability, utilizing main chain atoms from Ala^1^, Cys^2^, and Cys^30^ and the hydroxyl of Tyr^16^ (Fig. 3). The tetramer is further stabilized by a hydrogen bond between Arg^14^ and Tyr^21^ of one pair of subunits.

**Figure 5:**
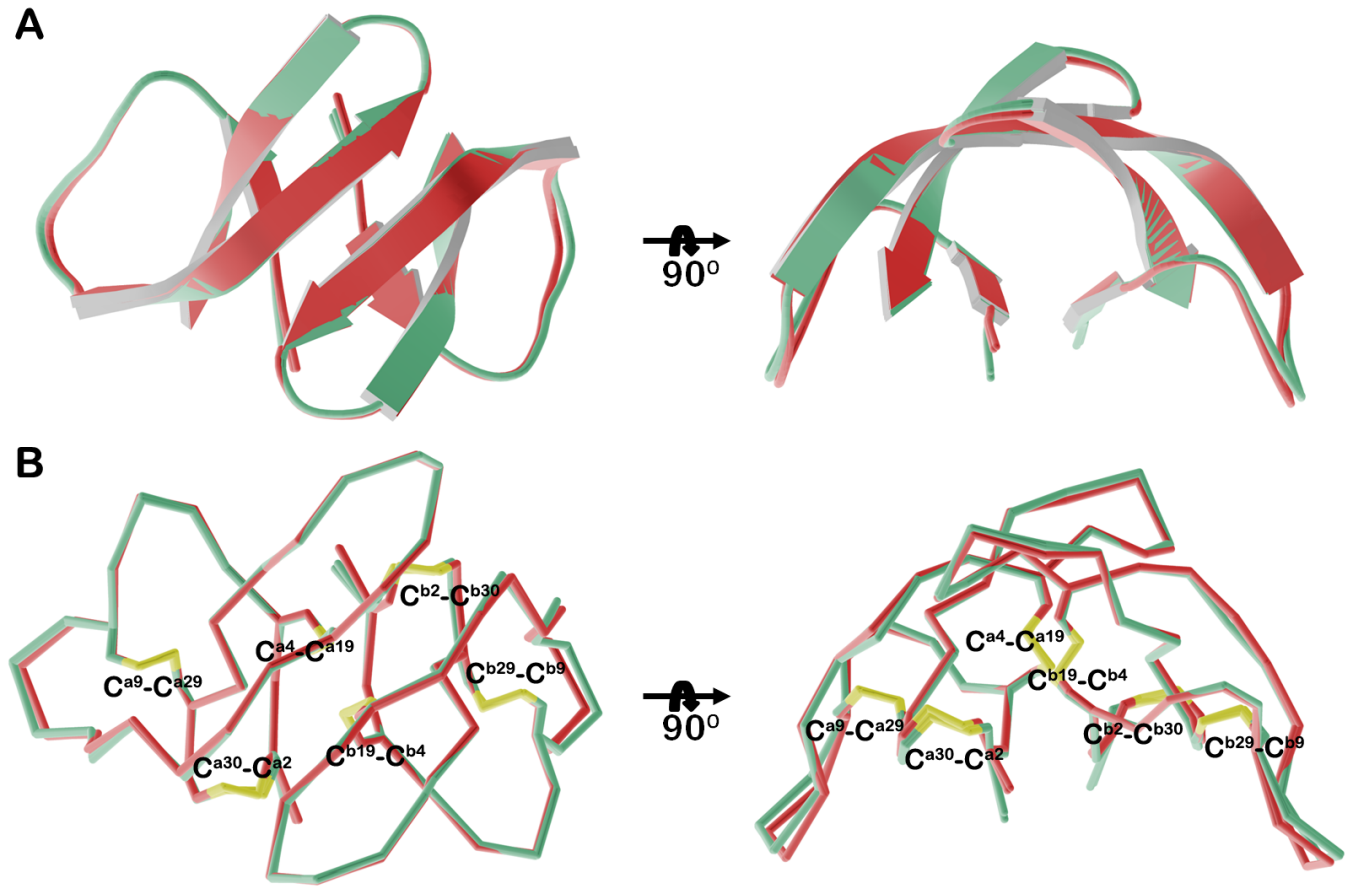
Y16A/F28A and Y16A/I20A/L25A/F28A-HNP1 dimers. (A) Ribbon and (B) α-carbon traces of all Y16A/F28A and Y16A/I20A/L25A/F28A HNP1 dimers present in the asymmetric unit of the crystal. Y16A/F28A dimers are in green and Y16A/I20A/L25A/F28A dimers in red. Disulfide bonds are shown in yellow. The RMSD is 0.216 Å between Y16A/F28A dimers and 0.093 Å between Y16A/I20A/L25A/F28A dimers.

**Figure 6:**
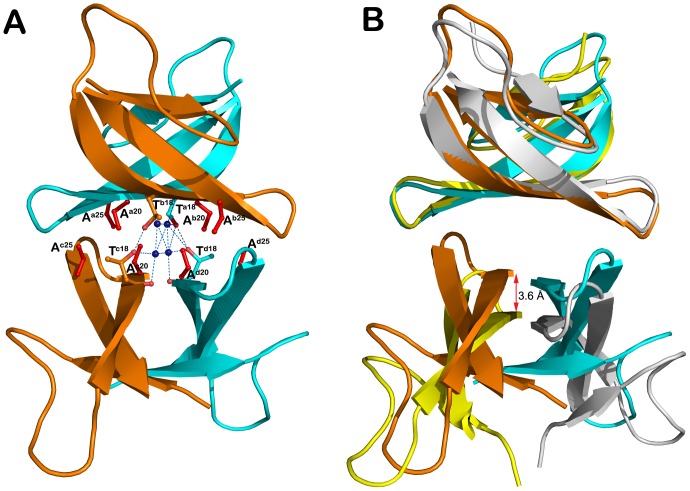
Quaternary structure of Y16A/I20A/L25A/F28A-HNP1. (A) Putative tetrameric assembly of Y16A/I20A/L25A/F28A-HNP1 in the crystal. Residues involved in tetramer formation are shown as balls and sticks with the mutated residues colored in red. The network of direct and water-mediated H-bonds formed at the tetramer interface is shown as blue dashes. (B) Comparison of Y16A/I20A/L25A/F28A-HNP1 and wild type HNP1 tetramers. Tetramers were aligned based on AB dimers and colored cyan and orange (Y16A/I20A/L25A/F28A-HNP1) and yellow and grey (HNP1, PDB:3GNY, [27] ). The red arrow indicates the shift of the CD dimer of Y16A/I20A/L25A/F28A-HNP1 relative to the CD dimer of wild-type HNP1.

Y16A/I20A/L25A/F28A-HNP1 forms a dimer which is very similar to the Y16A/F28A-HNP1 dimer (the average RMSDs between 60 equivalent Cα-atoms of 0.4 Å, Fig. 5). Solvent-accessible surface buried due to Y16A/I20A/L25A/F28A-HNP1 and Y16A/F28A-HNP1 dimer formation corresponds to 490 and 495 Å^2^ per monomer, respectively, indicating that these dimers are energetically indistinguishable. Surprisingly, although Y16A/I20A/L25A/F28A-HNP1 has mutated Ile^20^ and Leu^25^ that were previously identified as essential for wild type tetramer formation, analysis of intermolecular contacts within the crystal indicates that Y16A/I20A/L25A/F28A-HNP1 dimers arrange into a tetramer resembling closely the wild-type tetramer architecture (Fig. 6). Replacement of Ile^20^ and Leu^25^ side chains at the tetramer interface by less ‘bulky’ side chains of Ala allows Y16A/I20A/L25A/F28A-HNP1 dimers to pack more tightly together (Fig. 5B). The Y16A/I20A/L25A/F28A-HNP1 tetramer is stabilized through hydrophobic interactions involving Ala^20^ and a network of direct and water-mediated H-bonds formed between Thr^18^ and four water molecules that were ‘trapped’ at the tetramer interface (Fig. 5A). The buried interface area for Y16A/I20A/L25A/F28A-HNP1 tetramer formation is 1450 Å^2^ per dimer as compared to 1140 Å^2^ buried at the wild type HNP1 tetramer interface.

### Binding to HNP1 and gp120 as Determined by Surface plasmon Resonance

**Figure 7:**
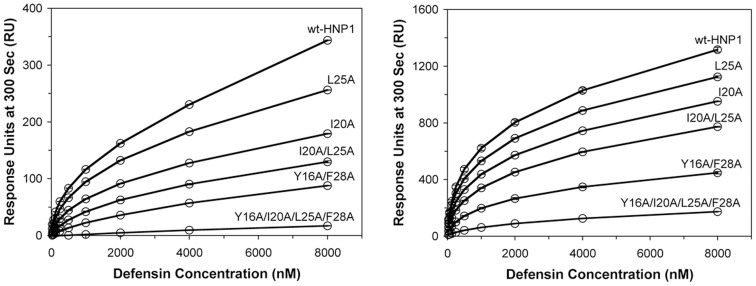
Surface plasmon resonance binding curves. Binding of HNP1 and mutants to 233 RU of HNP1 (left) and binding of HNP1 and mutants to 2770 RU of gp120 (right). The curves are plots of RU values at 300 s of association versus different defensin concentrations (from 0 nm to 8 µm). The RU values are the average readings from three measurements.

We tested the ability of HNP1 and its mutants to bind to immobilized HNP1 or to the HIV-1 protein gp120 by surface plasmon resonance. (Fig. 7) Against both the HNP1 and gp120 surfaces, response units were ranked HNP1> L25A>I20A>I20A/L25A>Y16A/F28A>Y16A/I20A/L25A/F28A. HNP1 bound to immobilized HNP1 with a 20-fold greater response at 8000 nM than Y16A/I20A/L25A/F28A-HNP1 bound to HNP1, and HNP1 bound to immobilized gp120 with an 8-fold greater response at 8000 nM than Y16A/I20A/L25A/F28A-HNP1 bound to gp120.

### Interactions of HNP1 Analogs with N36 Peptide

**Figure 8:**
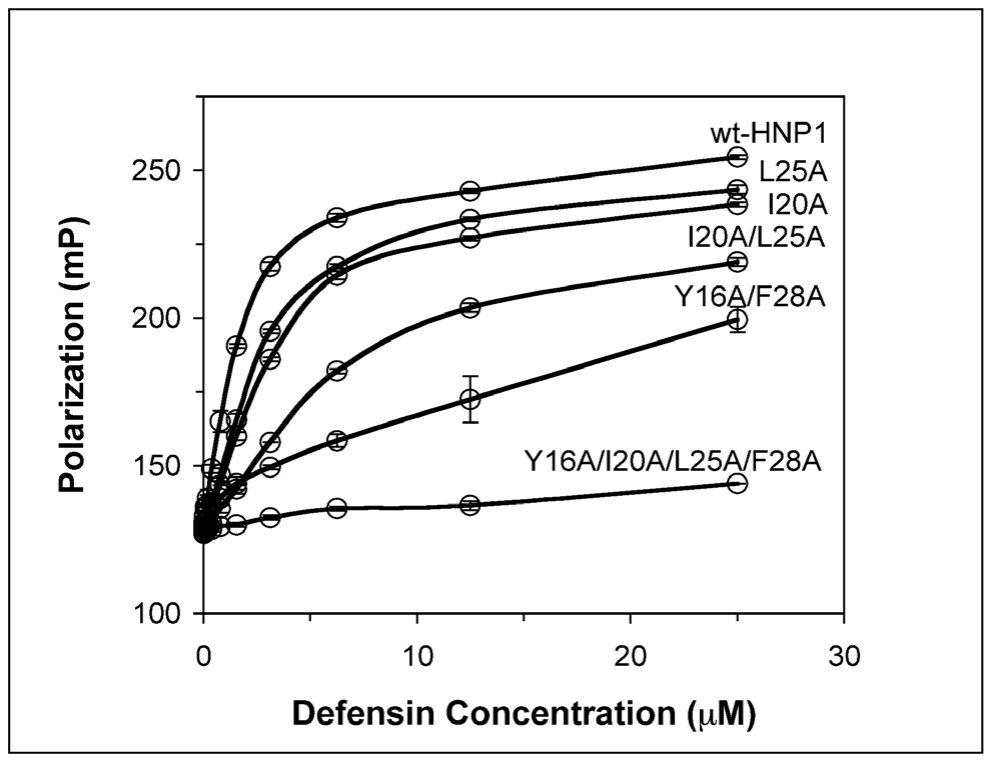
Defensin binding to N36 peptide as determined by fluorescence polarization. An increase in fluorescence polarization is indicative of defensin binding to fluorecently labeled N36 peptide. The curves are obtained from the averages of three measurements.

HNP1 inhibits HIV-1 infectivity via multiple mechanisms [50], [51]. Recently, HNP1 has been shown to be able to interact with the heptad repeat domains of of HIV-1 gp41 comprising both N36 and C34 peptides, and contribute to the inhibition of fusion by interfering with the formation of the fusogenic gp41 structure [52]. An N-acetyl-N36 peptide labeled with 6-carboxyfluorescein (FAM) at Lys^574^ (HIV gp160 numbering) at 50 nM in PBS was incubated at room temperature for 30 min with a 2-fold dilution series of HNP1 mutants (0.024–50 µM) before fluorescence polarization was measured in a triplicate assay on a Tecan Infinite M1000 multimode plate reader. All HNP1 analogs except Y16A/I20A/L25A/F28A-HNP1 bound N36 in a dose-dependent fashion, reflected by increasing fluorescence polarization values with the increase in defensin concentration. The binding of HNP1 analogs to N36 was also structure–dependent, since the polarization values were in the order HNP1> L25A>I20A>I20A/L25A>Y16A/F28A>Y16A/I20A/L25A/F28A at almost all of the concentration series. (Fig. 8).

### Inhibition of Lethal Factor by HNP1 Analogs

**Figure 9:**
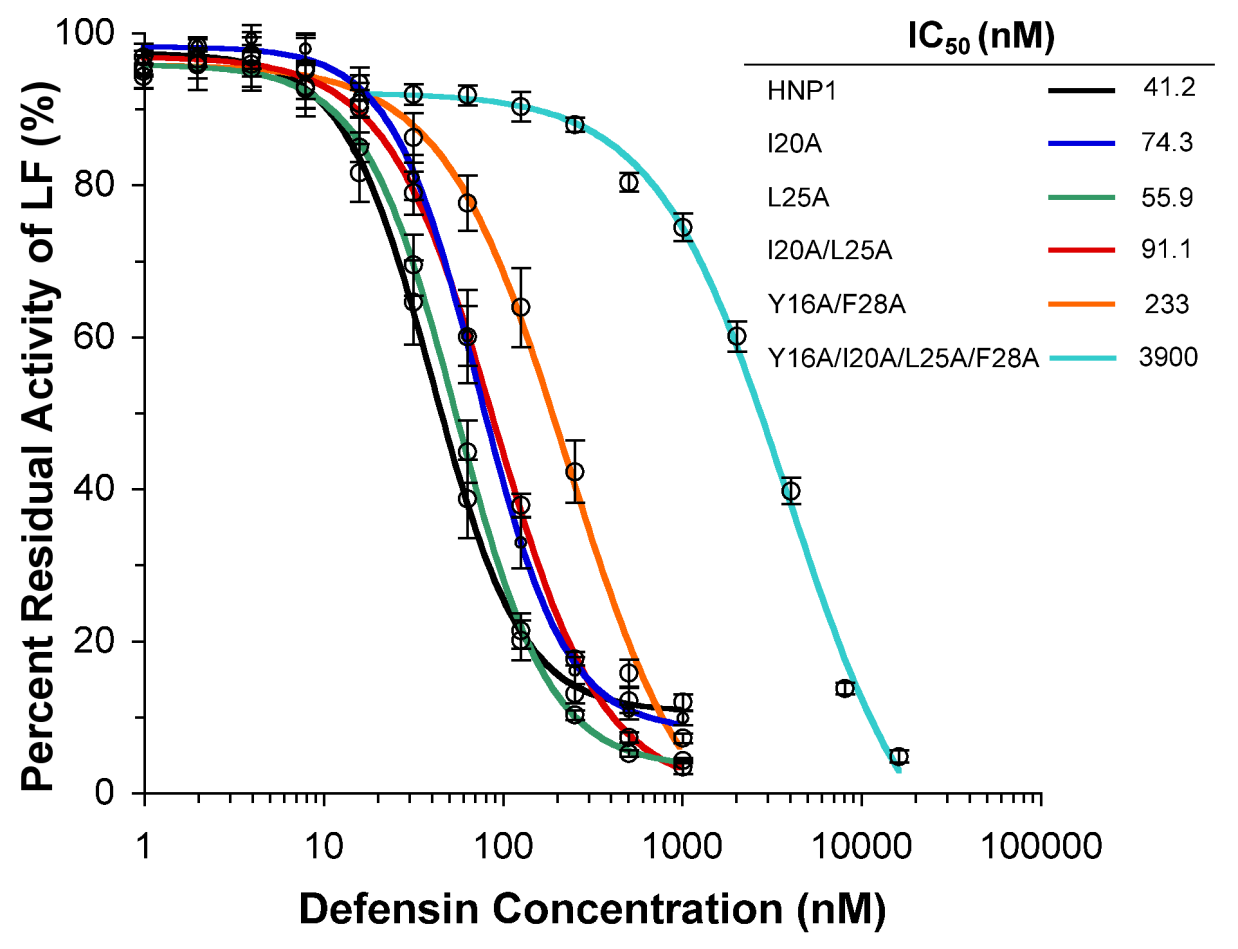
Inhibition of LF activity by different concentrations of HNP1 and mutant analogs. Each inhibition curve is the mean of three independent enzyme kinetic measurements except Y16AF28A-HNP1, which is the mean of duplicate measurements.

The inhibition of lethal factor by various defensins was quantified at 37°C using an enzyme kinetic assay [27]. As shown as inhibition curves and IC_50_ values in Fig. 9, replacement of Ile^20^ and/or Leu^25^ with Ala slightly weakened inhibition of LF relative to wild type HNP1. However, the inhibitory activity of Y16A/F28A and Y16A/I20A/L25A/F28A in particular was greatly suppressed with the latter yielding an IC_50_ 95-fold higher than that of wild type HNP1.

### Antibacterial Activity of HNP1 Analogs

**Figure 10:**
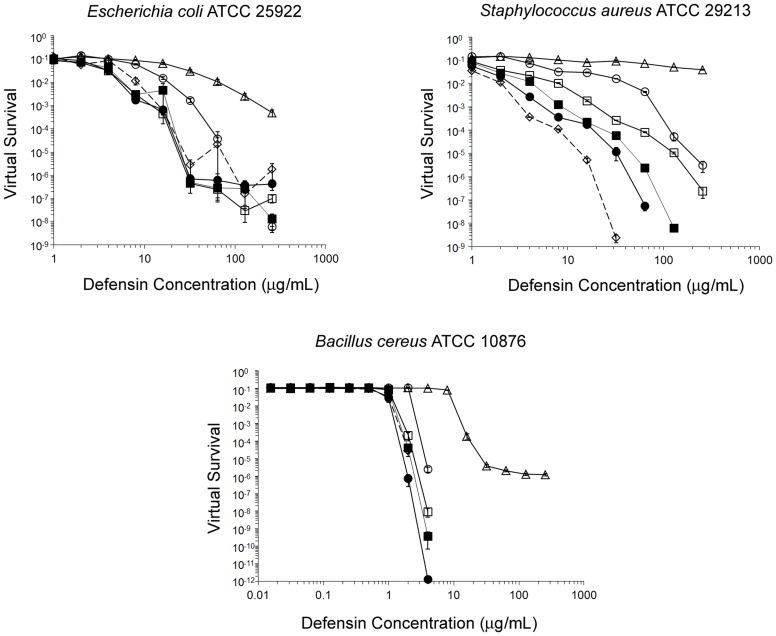
Antibacterial activity of defensins as determined by virtual colony count. *E. coli* ATCC 25922, *S. aureus* ATCC 29213 and *B. cereus* ATCC 10876 were exposed to HNP1 (◊; dotted line), I20A-HNP1 (▪), L25A-HNP1 (•), I20A/L25A-HNP1 (□), Y16A/F28A-HNP1 (○), and Y16A/I20A/L25A/F28A-HNP1 (Δ). Strains were exposed a twofold dilution series of defensins at concentrations varying from 1 to 256 µg/mL, except *B. cereus* was exposed to all defensins at 0.016 to 4 µg/mL and only Y16A/I20A/L25A/F28A-HNP1 at 1 to 256 µg/mL. Points equivalent to zero survival cannot be plotted on a logarithmic scale, such that against *S. aureus* virtual survival was zero above 32 µg/mL for HNP1, above 64 µg/mL for L25A-HNP1, and at 256 µg/mL for I20A-HNP1; against *B. cereus* virtual survival was zero at 4 µg/mL for HNP1. Each point is the mean of triplicate measurements, except HNP1 at 32 µg/mL against *S. aureus* (mean of two measurements; the third replicate gave a virtual survival value of zero) and L25A-HNP1 at 4 µg/mL against *B. cereus* (single measurement; the other two replicates gave virtual survival values of zero). Although error cannot easily be read directly from this plot, error values are quantified in Table 3.

Shown in Fig. 10 are virtual survival curves of *E. coli, S. aureus*, and *B. cereus* exposed to each defensin, and in Table 3 are the corresponding virtual lethal dose (vLD) values. Against *S. aureus*, the activity of all five HNP1 mutants was significantly undermined, giving activity in the rank order of HNP1> L25A>I20A>I20A/L25A>Y16A/F28A>Y16A/I20A/L25A/F28A at all defensin concentrations tested. By contrast, only the Y16A/F28A and Y16A/I20A/L25A/F28A mutations significantly reduced activity against *E. coli* and *B. cereus*. Notably, Y16A/I20A/L25A/F28A-HNP1 barely showed any activity at the maximum tested concentration of 256 µg/mL against *S. aureus*, whereas its activity remained less than vLD_99.9_ against *E. coli* and its vLD_99.9_ against *B. cereus* was 11-fold higher than HNP1. *S. aureus* growth curves were not necessarily parallel to non-defensin controls, suggesting that defensins can affect growth kinetics and delay threshold times after the addition of twice-concentrated Mueller-Hinton broth during the 12 h outgrowth phase of the virtual colony count assay.

**Table 3:** Antibacterial activities of HNP1 and mutants.

Test organism	vLD_50_ (µg/mL)					
	Wild-type HNP1	I20A	L25A	I20A/L25A	Y16A/F28A	I20A/L25A/Y16A/F28A
*E. coli*	4.23±0.80	3.22±0.50	2.78±0.19	2.89±0.10	8.65±0.24^a^	20.68±1.93^a^
*S. aureus*	<1	1.36±0.02^a^	1.13±0.06^b^	1.58±0.05^a^	5.48±0.26^a^	135.00±11.41^a^
*B. cereus*	0.78±0.08	1.03±0.03	0.77±0.12	1.04±0.05	2.09±0.01^a^	8.40±0.04^a^
	**vLD_90_ (µg/mL)**					
	**Wild-type HNP1**	**I20A**	**L25A**	**I20A/L25A**	**Y16A/F28A**	**I20A/L25A/Y16A/F28A**
*E. coli*	8.06±0.75	5.81±0.24	5.20±0.27	5.57±0.15	18.15±0.65^a^	64.92±8.68^a^
*S. aureus*	2.0±0.10	4.23±0.15^a^	2.54±0.05^a^	8.05±0.24^a^	41.32±0.96^a^	>256^a^
*B. cereus*	1.12±0.02	1.19±0.04	1.05±0.04	1.25±0.07	2.32±0.02^a^	10.06±0.16^a^
	**vLD_99_ (µg/mL)**					
	**Wild-type HNP1**	**I20A**	**L25A**	**I20A/L25A**	**Y16A/F28A**	**I20A/L25A/Y16A/F28A**
*E. coli*	13.92±1.41	13.85±2.58	13.05±1.81	11.94±2.07	33.41±0.45^a^	183.68±27.52^a^
*S. aureus*	3.25±0.03	8.60±0.45^a^	5.59±0.12^a^	19.72±0.51^a^	80.56±1.44^a^	>256^a^
*B. cereus*	1.40±0.03	1.44±0.08	1.21±0.06	1.60±0.14	2.69±0.04^a^	13.04±0.41^a^
	**vLD_99.9_ (µg/mL)**					
	**Wild-type HNP1**	**I20A**	**L25A**	**I20A/L25A**	**Y16A/F28A**	**I20A/L25A/Y16A/F28A**
*E. coli*	19.70±1.16	19.52±1.26	18.75±0.86	17.92±1.15	48.92±7.98	>256^a^
*S. aureus*	8.13±0.10	23.93±1.33^a^	18.37±0.69^a^	56.70±1.12^a^	115.40±5.43^a^	>256^a^
*B. cereus*	1.75±0.05	1.76±0.13	1.39±0.09^a^	1.95±0.20	3.12±0.06^a^	17.23±0.95^a^

Virtual lethal doses vLD_50_, vLD_90_, vLD_99_, and vLD_99.9_ are the concentrations that resulted in a survival of 0.5, 0.1, 0.01, and 0.001, respectively. Mean ± SEM is presented for triplicate measurements except where noted.

a Significantly different than wild-type HNP1 (paired t test, *p*<0.05).

b Average of two measurements; the third measurement was <1 µg/mL.

## Discussion

Defensins form a wide array of oligomeric structures. There is considerable structural diversity among the defensins, and the α-defensins are not the only group that has been shown to dimerize and multimerize. The authors of a study of the plant defensin NaD1 mutated a lysine to alanine and observed reduced activity, which they attributed to impaired dimer formation [53]. The activity could also have decreased due to a decrease in cationicity. This type of ambiguity does not apply to our present study, since we only mutated hydrophobic residues to alanine. The crystal structure of human β-defensin 2 depicted dimers and octamers, although the authors commented that it is unclear whether the octamer is physiologically relevant given the lack of conservation of the residues at the various subunit interfaces [54]. By contrast, a study of human β-defensins (HBD) 1–3 depicted HBD1 and 2 as monomers, while HBD3 crystallized as a dimer [55]. The enhanced activity of HBD3 compared to HBD1 and HBD2 may be related to its ability to dimerize. The θ-defensin retrocyclin-2 trimerized in solution, according to analytical ultracentrifugation and nuclear magnetic resonance results [56].

Starting with the crystal structure of HNP-3 [29], and the nuclear magnetic resonance (NMR) structures of HNP1 [57], [58], wild-type human α-defensin structures have typically depicted dimers. However, monomeric mutant forms of HNP1 and HD5 have been generated by N-methylating an amide bond at the dimer interface, thus breaking a hydrogen bond and introducing steric hindrance to the canonical dimerization scheme [26], [59]. In both cases, the monomeric mutant forms were as active against *E. coli* as the respective wild-type defensins. Other mutant forms have emphasized the versatility of the defensin framework. The HD5 canonical dimer with antiparallel β-strands existed in the crystal structure of the L29Abu-HD5 mutant, while the L29Nle-HD5 mutant exhibited a novel dimeric form with β-strands stacked in parallel [26]. Interestingly, the non-canonical L29Nle-HD5 molecule was functional, exhibiting less than wild-type activity against *S. aureus* but greater than wild-type activity against *E. coli* [26]. In the present study, we also discovered novel quaternary structures in the crystal, including a form of I20A-HNP1 that appears to be unable to form dimers or tetramers and a novel tetrameric form of I20A/L25A-HNP1. In addition, the canonical dimer formed by the quadruple mutant displayed a larger interface than wild-type. The HNP1 and HD5 results indicate that the presence of a canonical form of dimerization in the crystal does not correlate with α-defensin activity. Structural diversity may be a consequence of the small size of the peptides, leading to agile dynamic movements, combined with the rigidity of the tridisulfide structure that prevents major conformational transitions within the monomer. Defensin structural dynamics has been studied in solution in the context of binding to membranes or micelles [56], [60], [61]. The structure of HNP1 investigated using solid-state NMR suggested that the loop region between the first and second beta-strands is flexible and may change conformation when exposed to membranes [58]. However, the effect of membranes on HNP1 quaternary structure is poorly understood.

The stability of the dimer apparently varies from one α-defensin to the next. The HNP4 dimer is weakened by smaller hydrophobic side chains than HNP1 [62]. The HD5 dimer is stabilized by an additional short two-stranded beta sheet and hydrogen bond at the interface, yet dimerization is less important for HD5 than for HNP1 as indicated by the results of studies of forced monomeric defensins [26], [59]. However, a study of HD5 activity against non-enveloped viruses demonstrated that dimerization and multimerization are important for antiviral activity [63]. There is also diversity in modes of tetramerization among the α-defensins. The Ile^20^/Leu^25^-mediated HNP1 tetramer does not have an equivalent form in the cases of HNP4 or HD5, although there is evidence that HD5 forms tetramers [64]. Even though it has almost no direct antibacterial activity [22], HD6 exhibits the ability to form long chains of monomers [6], [62] and nanonets that trap bacteria and protect transgenic mice expressing HD6 from *Salmonella enterica* serovar Typhimurium infection [65].

Hydrophobicity has repercussions beyond oligomerization. In the case of HNP1, the finding that mutating four hydrophobic residues depicted in the crystal as contacting one another to alanine interferes with HNP1 function indicates that hydrophobicity at these positions is potentially evolutionarily significant. However, these results with HNP1 do not apply to α-defensins unviersally. Hydrophobicity is not conserved at any of the four positions (16, 20, 25, or 28) in an alignment of mammalian α-defensins. Radically different examples exist, such as the more basic rabbit neutrophil peptide NP-3a (Swiss-Prot identifier DEF1_RABIT), which has Ser at position 16 and Arg at positions 20, 25 and 28 (HNP1 numbering) [66]. Cationicity could be more significant than hydrophobicity in these cases, whereas for HNP1 and HD5 alanine scanning mutagenesis showed that hydrophobicity is clearly more important than cationicity [26], [28]. For HD5, positions 16, 20, 25, and 28 (HNP1 numbering; to translate into HD5 numbering add 1) are occupied by Ser, Glu, Leu and Leu, respectively, and the latter Leu, Leu^29^, has been shown to be the most important residue for antibacterial and LF-inhibiting activity [26]. A defensin like NP-3a would presumably form much different quaternary structures than HNP1, if any, because the electrostatic repulsion of the arginine side chains would make dimer and tetramer structures such as those described in the crystallographic section of this work unlikely.

The cumulative functional effect of the quadruple alanine mutations was similar to the effect of the single mutation, W26A [28]. This result sheds new light upon the role of Trp^26^, since interfering with this residue forming the hydrophobic core probably changes the conformation of several different side chains, including Phe^28^. The same study also showed that F28A had the second least SPR self-association of all HNP1 alanine scanning mutants, and the second lowest activity against *S. aureus* [28]. Interestingly, the F28A mutation did not prevent HNP1 from inhibiting LF and binding gp120, indicating that self-association and *S. aureus* activity are more sensitive to perturbations of HNP1 structure at this position. Here, our studies using the double and quadruple mutants that include the F28A mutation extend the previous findings. Unlike the F28A mutation alone, the Y16A/F28A double mutant and the quadruple mutant LF inhibition IC_50_ values increased 6-fold and 95-fold, respectively. In the virtual colony count assay, the double mutant Y16A/F28A-HNP1 had a vLD_99.9_ value against *S. aureus* 7-fold higher than HNP1, and Y16A/I20A/L25A/F28A-HNP1 failed to exhibit activity equivalent to the vLD_90_ level against *S. aureus*. The Y16A/F28A and Y16A/I20A/L25A/F28A mutants were also significantly less potent than HNP1 when assayed against both *E. coli* and *B. cereus*.

The functional consequences of the I20A and L25A mutations, alone or in concert, were milder than the Y16A/F28A double mutant or the quadruple mutant. LF inhibition was less than 2-fold different than wild-type for the single mutants and about 2-fold different for the I20A/L25A double mutant, as measured by IC_50_ values. In the virtual colony count assay, whereas the I20A and L25A mutations each made a significant difference against *S. aureus*, they did not make a significant difference against *E. coli* or *B. cereus* even in the I20A/I25A double mutant. Since Ile^20^ and Leu^25^ function as an isologous binding site for the formation of a dimer of dimers, their effect or lack thereof on *E. coli* is not surprising, given a previous study of N-methylated Ile^20^ that showed that dimerization is dispensible for *E. coli* activity [59]. The fact that the I20A/L25A double mutations were irrelevant, but the Y16A/F28A double mutations were quite relevant, for *E. coli* activity is insightful, because it demonstrates that the hydrophobicity of Tyr^16^ and Phe^28^ has repercussions beyond dimerization. If providing hydrophobic contacts for dimerization were the sole function of Tyr^16^ and Phe^28^, we would expect the activity of Y16A/F28A-HNP1 to be the same as wild-type HNP1, just as there was no difference between HNP1 or MeIle^20^-HNP1 when assayed against *E. coli* [59].

Functional assays correlated with each other and with hydrophobicity. Here we observed the binding affinity of HNP1 and mutants to HNP1 and gp120, as measured by SPR, and the N-terminus of HIV-1 gp41, as measured by fluorescence polarization. In all cases, binding was in agreement with LF inhibition and activity against *S. aureus*, in the order HNP1> L25A-HNP1> I20A-HNP1> I20A/L25A-HNP1> Y16A/F28A-HNP1> Y16A/I20A/L25A/F28A-HNP1. Correlation between these quantities was expected, given the previously observed correlation for mutants of Trp^26^ [28]. Self-association on the HNP1 surface was lessened by the mutations, reinforcing that these four positions are important for dimerization and multimerization. Diminished association with the two HIV-1 proteins also suggests that dimerization and multimerization are important for antiviral function. However, the diversity of monomeric, dimeric and tetrameric crystallographic forms suggest that propensity to form certain quaternary structures in the crystal does not always inform or predict defensin function.

Although these crystallographic results do not support the premise that crystal structures are necessarily uniformly functionally insightful, the convergence of evidence from the wide variety of other methods employed has yielded the ability to rank the contribution of each of the side chains mutated to alanine. According to the Wimley-White experimentally determined hydrophobicity scales for proteins at membrane interfaces [67], the five most hydrophobic residues are Trp>Phe>Tyr>Leu>Ile. Our studies of HNP1 have determined the relative importance of residues to be the same, save for the transposition of Ile and Leu: Trp^26^>Phe^28^> Tyr^16^> Ile^20^> Leu^25^. The Wimley-White scale was determined using large unilamellar vesicles comprised of the zwitterionic phospholipid palmitoyloleoylphosphatidylcholine; the scale might have differed had they used more anionic membranes typical of bacteria. Nevertheless, the results of our alanine scanning mutagenesis studies of HNP1 with single, double, and quadruple alanine mutants emphasize the importance of hydrophobicity as the primary factor that determines activity, and indicate that the importance of a residue is roughly proportional to its hydrophobicity regardless of structural location.

Although these residues are shown to be at oligomeric interfaces in most crystal structures, the consequences of decreasing hydrophobicity at any of these positions on crystallographic results indicates that the canonical dimer is delicate. In the dynamic environment of the liquid phase as opposed to the solid crystal, a loose association between monomers may allow hydrophobic residues to exchange their affinities for one another for interactions with carbon atoms in diverse targets such as bacterial membranes, enzymes and proteins. Weak van der Waals interactions can be interchangeable and do not necessarily result in specificity. Therefore, while the significance of these four residues for function has been clearly established, the degree to which oligomers such as those implied by the canonical dimer contribute to defensin activity remains an open question worthy of further study.

## Supporting Information

Figure S1 **Stereo view of the backbone (A) and ribbon (B) traces of superimposed HNP1 mutant monomers with the monomers of wild type HNP1 (PDB:3GNY,** [**27**] **).** Wild type HNP1 is shown in green, I20A-HNP1 in turquoise, Y16A/F18A-HNP1 in gold, I20A/L25A-HNP1 in violet, and Y16A/I20A/L25A/F28A-HNP1 in coral. Disulfide bonds are shown in yellow, with sulfurs in yellow, nitrogens in blue, and oxygens in red. The crystal of the I20A-HNP1 mutant contained two defensin molecules in the asymmetric unit, and the I20A/L25A, Y16A/F28A and Y16A/I20A/L25A/F28A mutants crystallized with four defensin molecules in the asymmetric unit. Pairwise superposition of the crystallographically independent copies of I20A-HNP1, I20A/L25A-HNP1, Y16A/F28A-HNP1 and Y16A/I20A/L25A/F28A-HNP1 yielded average Cα RMDS values of 0.09, 0.66, 0.20, and 0.10 Å for 30 atoms, respectively.
(TIF)

Figure S2 **Quaternary structure of Y16AF28A-HNP1.** (A) Stereo view of the structural alignment of the Y16A/F28A-HNP1 and F28A-HNP1 dimers. Dimers were aligned based on monomer A and colored cyan and green (Y16A/F28A-HNP1) and pink and grey (F28A-HNP1, PDB:3LOE, [28]). Both dimers are stabilized by the same network of main chain H-bonds (shown as blue dashes). The H-bond formed between hydroxyl groups of Tyr^3^ in the F28A-HNP1 dimer (shown as magenta dashes) is replaced by the H-bond formed between the main chain nitrogen of Ala^1^ and the hydroxyl of Tyr^3^ in Y16A/F28A-HNP1 dimer (shown as light blue dashes). The molecular surface buried within the Y16A/F28A-HNP1 dimer is 494 Å^2^ per monomer, which compares to 500 Å^2^ for the F28A-HNP1 dimer. (B) Structural alignment of Y16A/F28A-HNP1 and wild type HNP1 tetramers. Tetramers were aligned based on the AB dimer and residues involved in tetramer formation are shown as balls and sticks. The Y16A/F28A-HNP1 dimers are colored as in (A) and the HNP1 dimers (PDB:3GNY, [27]) are colored red and blue. The molecular surface buried within the Y16A/F28A-HNP1 tetramer is 1420 Å^2^ per dimer, which compares to 1140 Å^2^ for the HNP1 tetramer.
(TIF)
