# Supplementary figures and images for: Single, Double and Quadruple Alanine Substitutions at Oligomeric Interfaces Identify Hydrophobicity as the Key Determinant of Human Neutrophil Alpha Defensin HNP1 Function
Source: PLoS One. 2013 Nov 13;8(11):e78937. doi: 10.1371/journal.pone.0078937 (PMC3827289; doi:10.1371/journal.pone.0078937)

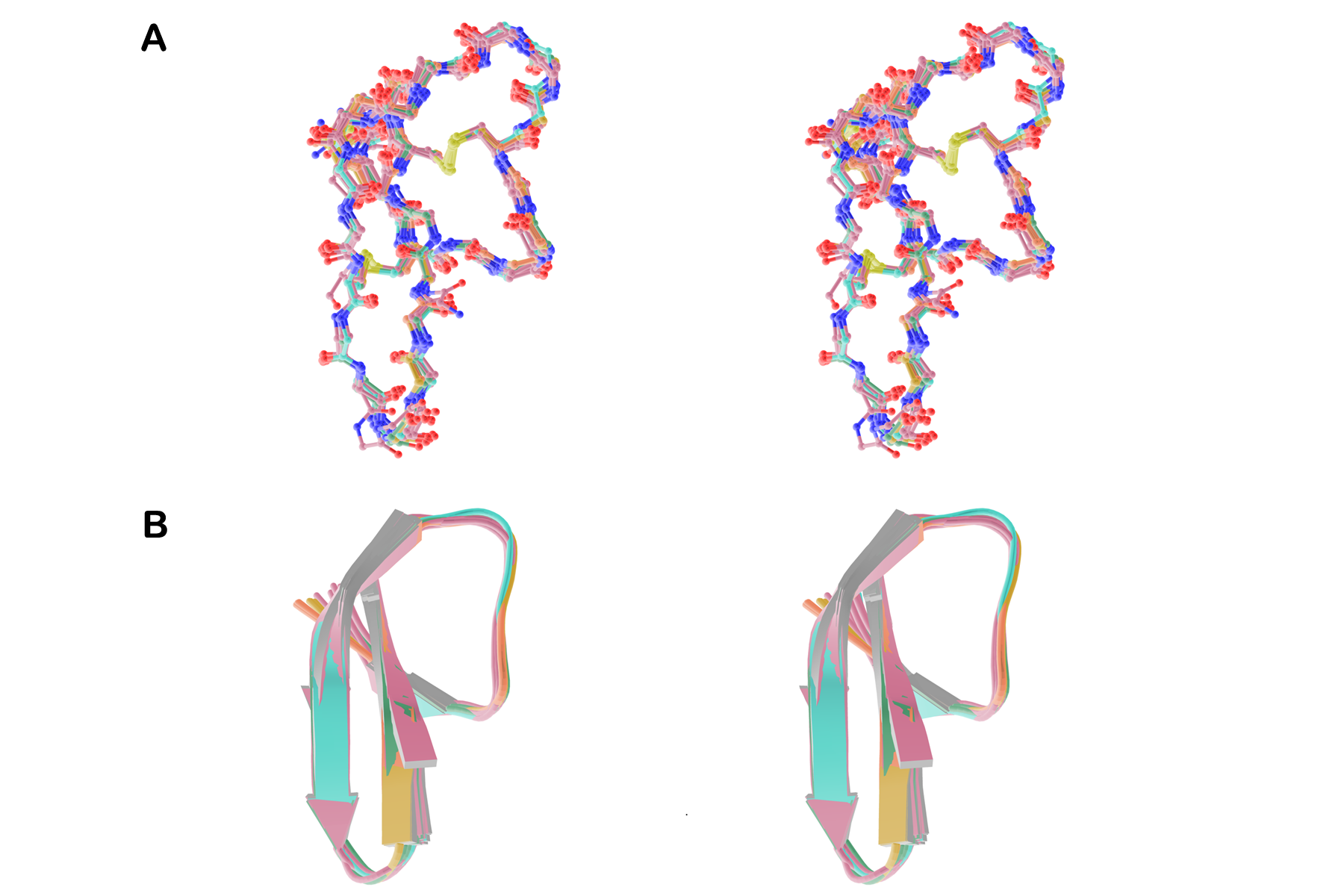

Supplement: Figure S1 — Stereo view of the backbone (A) and ribbon (B) traces of superimposed HNP1 mutant monomers with the monomers of wild type HNP1 (PDB:3GNY, [27] ). Wild type HNP1 is shown in green, I20A-HNP1 in turquoise, Y16A/F18A-HNP1 in gold, I20A/L25A-HNP1 in violet, and Y16A/I20A/L25A/F28A-HNP1 in coral. Disulfide bonds are shown in yellow, with sulfurs in yellow, nitrogens in blue, and oxygens in red. The crystal of the I20A-HNP1 mutant contained two defensin molecules in the asymmetric unit, and the I20A/L25A, Y16A/F28A and Y16A/I20A/L25A/F28A mutants crystallized with four defensin molecules in the asymmetric unit. Pairwise superposition of the crystallographically independent copies of I20A-HNP1, I20A/L25A-HNP1, Y16A/F28A-HNP1 and Y16A/I20A/L25A/F28A-HNP1 yielded average Cα RMDS values of 0.09, 0.66, 0.20, and 0.10 Å for 30 atoms, respectively. (TIF) [file pone.0078937.s001.tif]

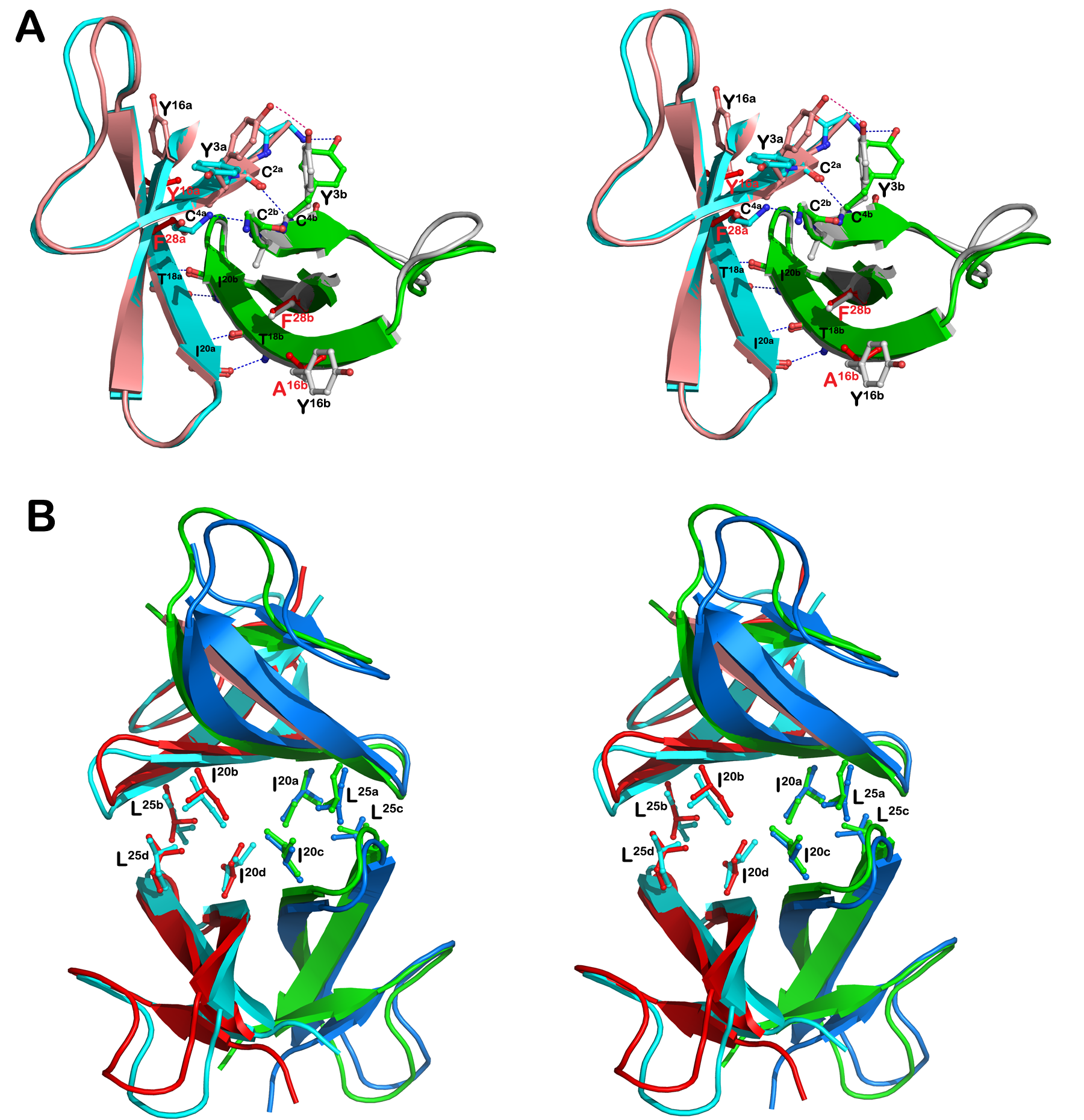

Supplement: Figure S2 — Quaternary structure of Y16AF28A-HNP1. (A) Stereo view of the structural alignment of the Y16A/F28A-HNP1 and F28A-HNP1 dimers. Dimers were aligned based on monomer A and colored cyan and green (Y16A/F28A-HNP1) and pink and grey (F28A-HNP1, PDB:3LOE, [28]). Both dimers are stabilized by the same network of main chain H-bonds (shown as blue dashes). The H-bond formed between hydroxyl groups of Tyr3 in the F28A-HNP1 dimer (shown as magenta dashes) is replaced by the H-bond formed between the main chain nitrogen of Ala1 and the hydroxyl of Tyr3 in Y16A/F28A-HNP1 dimer (shown as light blue dashes). The molecular surface buried within the Y16A/F28A-HNP1 dimer is 494 Å2 per monomer, which compares to 500 Å2 for the F28A-HNP1 dimer. (B) Structural alignment of Y16A/F28A-HNP1 and wild type HNP1 tetramers. Tetramers were aligned based on the AB dimer and residues involved in tetramer formation are shown as balls and sticks. The Y16A/F28A-HNP1 dimers are colored as in (A) and the HNP1 dimers (PDB:3GNY, [27]) are colored red and blue. The molecular surface buried within the Y16A/F28A-HNP1 tetramer is 1420 Å2 per dimer, which compares to 1140 Å2 for the HNP1 tetramer. (TIF) [file pone.0078937.s002.tif]
